# Coronary Angioplasty with Drug-Coated Balloons: Pharmacological Foundations, Clinical Efficacy, and Future Directions

**DOI:** 10.3390/medicina61081470

**Published:** 2025-08-15

**Authors:** Valentin Chioncel, Flavius Gherasie, Alexandru Iancu, Anamaria-Georgiana Avram

**Affiliations:** 1 Department of Cardiology, University of Medicine and Pharmacy Carol Davila, 050474 Bucharest, Romania; flavius.gherasie@umfcd.ro; 2 Emergency Clinical Hospital Dr. Bagdasar-Arseni, 041915 Bucharest, Romania; alx_med@yahoo.com (A.I.); anamaria.g.avram@gmail.com (A.-G.A.)

**Keywords:** drug-coated balloons, percutaneous coronary intervention, paclitaxel, sirolimus, in-stent restenosis, small vessel disease, de novo lesions

## Abstract

Drug-coated balloons (DCBs) have transformed percutaneous coronary intervention (PCI) by delivering antiproliferative drugs directly to the arterial wall, offering a stent-less approach that mitigates the risks associated with permanent metallic implants. Initially developed for in-stent restenosis (ISR), DCBs have demonstrated robust efficacy in reducing neointimal hyperplasia and target lesion revascularization (TLR) rates across diverse coronary lesions, including small vessel disease (SVD), de novo lesions, and complex anatomies such as bifurcation lesions. Paclitaxel-coated balloons have long been the cornerstone of DCB therapy due to their established clinical outcomes, but sirolimus-coated balloons are emerging as a promising alternative with potentially superior safety profiles and sustained drug release. The pharmacological mechanism of DCBs relies on rapid drug transfer during brief balloon inflation, achieving high local concentrations without residual foreign material. Landmark trials, such as BASKET-SMALL 2, RESTORE SVD, and AGENT IDE, have demonstrated comparable or non-inferior outcomes of DCBs versus drug-eluting stents (DESs) in specific settings, with lower rates of stent thrombosis and shorter dual antiplatelet therapy (DAPT) requirements. Despite these advances, challenges persist, including optimizing drug formulations, ensuring uniform delivery, and addressing calcified lesions. Ongoing research into novel coatings, dual–drug systems, and artificial intelligence (AI)-guided interventions is poised to redefine PCI strategies. This review provides a comprehensive analysis of drug-coated balloon (DCB) angioplasty, not limited to specific clinical scenarios such as in-stent restenosis, small vessel disease, or bifurcation lesions, highlighting their transformative role in coronary artery disease (CAD) management. Instead, it addresses the full spectrum of pharmacological principles, mechanisms of action, clinical indications, comparative efficacy across various coronary artery disease contexts, and future directions of DCBs.

## 1. Introduction

The management of coronary artery disease (CAD) has evolved dramatically since Andreas Grüntzig introduced balloon angioplasty in 1977 [1]. While revolutionary, early balloon angioplasty was limited by elastic recoil, abrupt vessel closure, and restenosis rates as high as 30–50% [2,3]. These challenges prompted the development of bare-metal stents (BMSs) in the late 1980s, which provided mechanical scaffolding to maintain vessel patency [4,5]. However, BMS introduced neointimal hyperplasia, leading to in-stent restenosis (ISR) in 20–30% of cases [6]. The advent of drug-eluting stents (DESs) in the early 2000s, which released antiproliferative agents to inhibit smooth muscle cell proliferation, reduced restenosis rates to below 10% [7]. Despite their efficacy, DESs are associated with delayed endothelialization, late stent thrombosis, and the need for prolonged dual antiplatelet therapy (DAPT), increasing bleeding risks [8].

**Figure 1:**
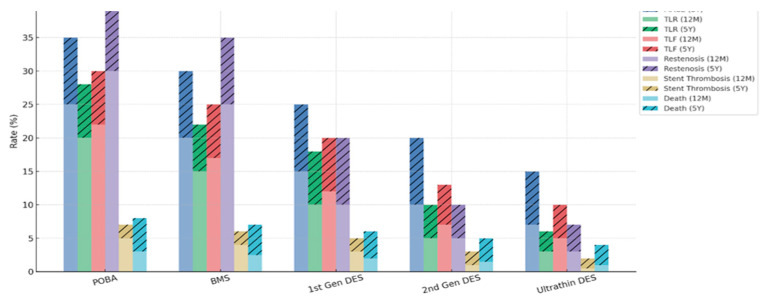
Graphical comparison of clinical outcomes at 12 months vs. 5 years across PCI techniques.

While the introduction of bare-metal stents (BMSs) and first-generation DESs dramatically reduced restenosis and target lesion revascularization (TLR) compared to POBA, the long-term outcomes such as MACE and stent thrombosis remained suboptimal. Subsequent evolution toward second-generation and ultrathin DES further improved safety and efficacy, yet certain limitations persist, particularly in small vessels, bifurcations, and high-bleeding-risk populations (Figure 1).

Despite advancements in DES technology, the presence of a permanent metallic scaffold remains a central limitation, associated with delayed vascular healing, persistent inflammation, and late thrombotic events. These observations have revived interest in non-scaffold-based therapies, particularly drug-coated balloons (DCBs), which deliver antiproliferative agents locally without leaving behind foreign material.

DCBs have demonstrated promising results, especially in in-stent restenosis (ISR), small vessel disease, and specific high-risk anatomical settings. The absence of permanent implants with DCBs allows for the preservation of native vasomotion and may reduce long-term complications such as very late stent thrombosis or neoatherosclerosis. In this context, the integration of DCBs into the PCI armamentarium represents a paradigm shift, moving from permanent scaffolding toward transient drug delivery with long-lasting vascular benefit.

Drug-coated balloons (DCBs) emerged as an innovative solution to address these limitations, embodying a leave-nothing-behind philosophy. By delivering antiproliferative drugs directly to the vessel wall during brief balloon inflation, DCBs avoid permanent implants, reducing risks of chronic inflammation, neoatherosclerosis, and stent-related complications [9]. DCBs also enable shorter DAPT durations, a critical advantage for patients at a high bleeding risk (HBR) [10].

Initially developed for ISR, DCBs have expanded to small vessel disease (SVD), de novo lesions, and complex anatomies, supported by robust clinical evidence [11,12]. This review provides an in-depth analysis of the pharmacological foundations, clinical applications, and future directions of DCBs in PCI, emphasizing their role in modern cardiology.

Thus, while DES remains the cornerstone in most PCI scenarios, DCBs offer a complementary strategy, especially where long-term outcomes require the minimization of metal burden and improved vessel healing. Future trials will further define their optimal role.

## 2. Advantages of Drug-Coated Balloons Compared to Stents

DESs, introduced earlier, provide a permanent metallic scaffold that prevents acute vessel closure and significantly reduces restenosis by delivering antiproliferative drugs over time. Their primary advantages include long-term vessel patency and effectiveness in complex or long lesions, making them a cornerstone for many procedures. However, DESs carry disadvantages such as the risk of late stent thrombosis, necessitating extended dual antiplatelet therapy (DAPT), and potential for in-stent restenosis or neoatherosclerosis, adding procedural and follow-up complexity.

In contrast, drug-coated balloons (DCBs), a more recent innovation, offer a distinct therapeutic benefit by delivering antiproliferative agents directly to the vessel wall without leaving a permanent implant, thereby addressing several limitations associated with drug-eluting stents (DESs). Their key advantages include reduced long-term risks like thrombosis, no need for prolonged DAPT, and suitability for small vessels, bifurcation lesions, or in-stent restenosis cases where scaffolds are less ideal. This makes DCBs a flexible option, particularly in patients with contraindications to long-term anticoagulation.

Drug-coated balloons (DCBs) are increasingly utilized in percutaneous coronary intervention (PCI) for specific patient profiles, particularly in-stent restenosis (ISR), small vessel disease (SVD), and high bleeding risk (HBR) cases. Selection criteria optimize outcomes by targeting lesions and patients where DCBs offer advantages over drug-eluting stents (DESs), such as shorter dual antiplatelet therapy (DAPT) and no permanent implant.

One of the primary advantages of DCBs lies in their ability to maintain vascular physiology. By avoiding the implantation of a metallic scaffold, DCBs allow for natural arterial healing while preserving vessel elasticity and function. In contrast, DESs permanently alter the vessel environment and may impair vasomotion.

A key clinical benefit of DCBs is the reduced risk of late and very late stent thrombosis by avoiding permanent implants. Unlike first-generation DESs, which delay healing and cause inflammation, DCBs offer a safer long-term profile in selected patients.

DCBs also enable a shorter duration of dual antiplatelet therapy (DAPT), which is especially valuable in patients at high bleeding risk. The absence of a permanent implant reduces the need for extended DAPT, thereby minimizing bleeding complications without compromising efficacy in restenosis prevention.

Moreover, DCBs are particularly advantageous in anatomically challenging scenarios such as small vessel disease and bifurcation lesions (especially Medina 0-0-1). Stenting in small arteries is often associated with higher restenosis rates, and bifurcations may require complex strategies to protect side branches. DCBs deliver uniform drug distribution without mechanical limitations, making them an attractive option in these contexts.

As a stent-free modality with comparable efficacy, DCBs represent a significant advancement in percutaneous coronary intervention (PCI). Emerging data, including results from the BASKET-SMALL 2 and PICCOLETO II trials, support the role of DCBs in de novo lesions, demonstrating similar long-term outcomes and lower revascularization rates compared to DESs [13,14].

However, DCBs have drawbacks, including the need for precise deployment to ensure adequate drug transfer and a potential for higher restenosis rates due to the absence of a scaffold, which can limit their efficacy in certain anatomies or unstable lesions.

Drug-coated balloons may also offer significant advantages over drug-eluting stents in patients with hypersensitivity to metals, a concern in percutaneous coronary intervention due to potential adverse reactions to stent materials like nickel or cobalt. DCBs deliver antiproliferative drugs to the vessel wall without leaving a permanent metallic scaffold, mitigating risks associated with metal hypersensitivity, such as chronic inflammation, in-stent restenosis (ISR), and late stent thrombosis.

Metal hypersensitivity can trigger local inflammatory responses, contributing to neoatherosclerosis and ISR [15,16]. DESs, despite advancements, retain a metallic structure that may provoke such reactions, particularly in sensitive patients. A comprehensive review notes that DCBs avoid “allergy to metal or polymer,” reducing chronic inflammation and preserving vessel anatomy. The absence of a permanent implant eliminates the risk of long-term foreign body reactions, which can exacerbate hypersensitivity-related complications.

Clinical trials support DCBs’ efficacy in settings where metal avoidance is critical. The PACCOCATH ISR-I and -II trials demonstrated that paclitaxel-coated DCBs significantly reduced late lumen loss (LLL) and major adverse cardiovascular events (MACE) compared to plain balloon angioplasty in BMS-ISR, with no metal-related complications [17].

Similarly, the PEPCAD II trial showed DCBs outperforming paclitaxel-eluting stents in LLL reduction, suggesting effective drug delivery without a metallic scaffold [18].

For de novo lesions, the DEBUT trial (2020) found DCBs noninferior to bare-metal stents (BMSs) in high-bleeding-risk patients, with no hypersensitivity-related adverse events reported, highlighting their safety in avoiding metal implants. This trial showed that DCBs reduced bleeding events (4.1% vs. 9.2%; *p* = 0.04) compared to bare-metal stents in HBR patients [19]. Suitable profiles include those with HBR scores ≥ 25 or comorbidities like renal failure.

Sirolimus DCBs show promise but require further validation [17]. Patients with complex lesions (e.g., bifurcations) or acute coronary syndromes may benefit, though evidence is evolving [20].

Thus, DCBs provide a compelling alternative to stents by eliminating metal-related hypersensitivity risks, reducing inflammation, and enabling shorter DAPT. Ongoing trials like TRANSFORM II [21] will further clarify their role, but current evidence strongly supports DCBs for patients with metal hypersensitivity undergoing PCI.

## 3. Pharmacological Premises

(a) Drug Selection and Mechanism of Action

DCBs deliver antiproliferative agents to the arterial wall during 30–60 s of balloon inflation, inhibiting neointimal hyperplasia without permanent scaffolds [9]. The two primary drugs used are paclitaxel and sirolimus, each with distinct pharmacological profiles. Paclitaxel exerts its antiproliferative effect by stabilizing microtubules and inducing cell cycle arrest at the G_2_/M phase, thereby preventing mitosis and promoting apoptosis [9,15]. Its high lipophilicity enables rapid uptake and prolonged retention in the arterial wall following balloon inflation, ensuring sustained pharmacologic activity [22]. In contrast, sirolimus inhibits the mTOR pathway, leading to cell cycle arrest in the G_1_ phase, and requires advanced excipient technologies to ensure adequate transfer and retention in the vessel wall [17,23,24].

**Paclitaxel**, a cytotoxic diterpenoid, binds to the subunit of tubulin, stabilizing microtubules and arresting cell division in the G2/M phase [25]. Its high lipophilicity (logP 3.96) enables rapid tissue uptake and prolonged retention, with tissue concentrations detectable for weeks after a single application [11]. This property makes paclitaxel ideal for DCBs, as it ensures sustained antiproliferative effects despite brief exposure. However, its cytotoxic nature can delay endothelial healing, potentially increasing inflammation in some cases [11,25].

**Figure 2:**
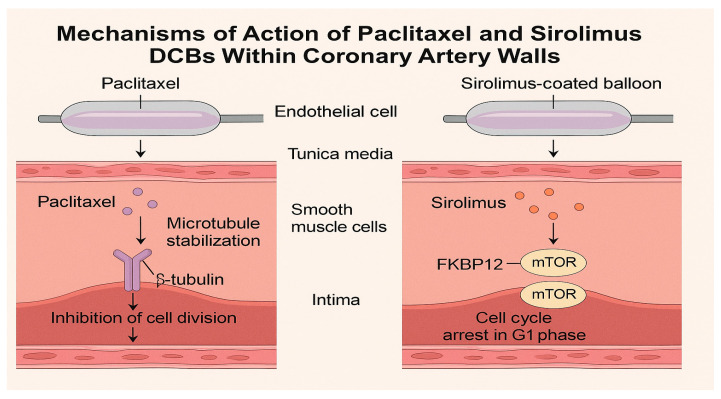
Mechanisms of action of paclitaxel and sirolimus drug-coated balloons (DCBs) within coronary artery walls.

**Sirolimus**, a cytostatic macrolide, inhibits the mammalian target of rapamycin (mTOR) pathway by binding to FKBP12, arresting the cell cycle in the G1 phase [26]. With lower lipophilicity (logP 2.5) and a shorter tissue half-life, sirolimus requires advanced delivery systems to achieve sustained effects [25]. Recent innovations, including phospholipid carriers and micro-reservoir formulations, enhance sirolimus transfer and retention, improving its efficacy in DCBs [12]. Preclinical studies demonstrate that sirolimus promotes faster endothelialization and reduces inflammatory responses compared to paclitaxel, suggesting a superior healing profile (Figure 2) [26].

(b) Drug Delivery Technologies and Excipient Systems

The efficacy of DCBs hinges on the excipient matrix, which facilitates drug transfer from the balloon surface to the arterial wall.

Paclitaxel-coated balloons commonly use excipients such as shellac, urea, iopromide, or contrast agents to enhance solubility and adhesion [9]. These form a thin film that enables rapid drug release upon inflation, achieving high local concentrations (up to 500 μg/g tissue) within seconds [11].

**Figure 3:**
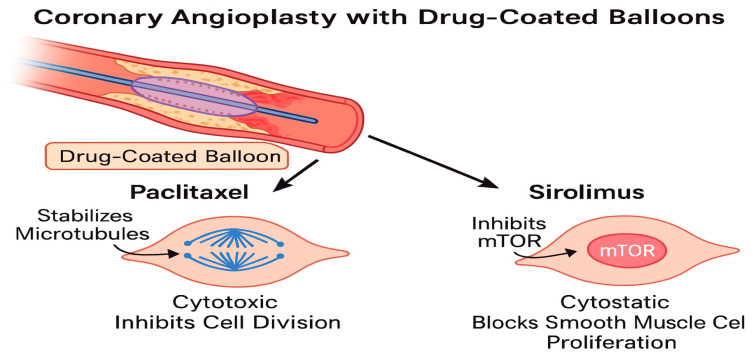
Distinct cellular mechanisms of paclitaxel and sirolimus during coronary angioplasty with DCBs.

Sirolimus-coated balloons require more complex excipients due to their lower lipophilicity and larger molecular size (MW 914 Da vs. 853 Da for paclitaxel) (Figure 3).

Formulations such as butyryl-tri-n-hexyl-citrate (BTHC), polylactic-co-glycolic acid-PLGA), and bioresorbable phospholipids sustain drug release and improve vascular uptake [12]. For example, the Selution SLR drug-coating balloon uses a micro-reservoir system to maintain sirolimus release for up to 60 days [27]. Table 1 summarizes key drug and excipient properties.

Although drug-coated balloons (DCBs) emerged later than stents, including drug-eluting stents (DESs), both have established unique roles in percutaneous coronary intervention (PCI), reflecting evolving cardiovascular care.

## 4. Clinical Applications and Indications of DCBs

DCBs are indicated for a range of coronary lesions where stents are suboptimal or contraindicated, including ISR, SVD, de novo lesions, bifurcation lesions, and HBR patients. Their versatility stems from their ability to deliver drugs without permanent implants, preserving vessel physiology and reducing long-term complications [24].

(a) In-Stent Restenosis (ISR)

ISR, caused by neointimal proliferation following stent deployment, remains a primary indication for DCBs. Re-treating ISR with additional stents increases metal burden, procedural complexity, and restenosis risk [12,28,29]. Landmark trials, including PEPCAD II [18], ISAR-DESIRE 3 [10], and RIBS IV [30], have established DCBs as non-inferior or superior to repeat DES for both BMS- and DES-ISR. The DAEDALUS meta-analysis (*n* = 2110) confirmed comparable target lesion failure (TLF) rates (12.2% vs. 13.5%) and lower bleeding complications with DCBs due to shorter DAPT durations [31].

The AGENT IDE trial is a pivotal, multicenter, randomized study evaluating the efficacy and safety of a paclitaxel-coated balloon (DCB) compared to an uncoated balloon in patients with coronary in-stent restenosis (ISR) undergoing percutaneous coronary intervention (PCI). Conducted across 40 U.S. centers, 600 patients were randomized 2:1 to receive either a paclitaxel-coated balloon (*n* = 406) or an uncoated balloon (*n* = 194) [32].

**Figure 4:**
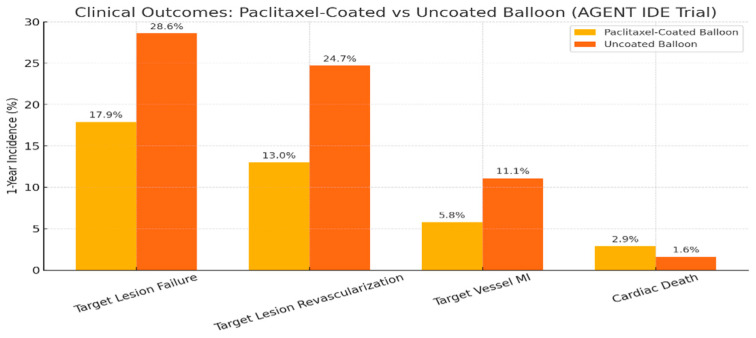
One year clinical outcomes: paclitaxel-coated vs. uncoated balloon.

At 1-year follow up, the primary composite endpoint—target lesion failure (TLF), including ischemia-driven target lesion revascularization (TLR), target vessel myocardial infarction (MI), or cardiac death—occurred significantly less in the paclitaxel group (17.9%) versus the uncoated group (28.6%), meeting superiority criteria (HR 0.59, 95% CI 0.42–0.84, *p* = 0.003). TLR was also significantly lower (13.0% vs. 24.7%, HR 0.50, *p* = 0.001), as was target vessel MI (5.8% vs. 11.1%, HR 0.51, *p* = 0.02). Cardiac death rates did not differ significantly (Figure 4).

Notably, there were no cases of definite/probable stent thrombosis in the paclitaxel group compared to six (3.2%) in the uncoated group (*p* < 0.001). Subgroup analysis showed consistent benefit across key populations, including those with multiple stent layers and diabetes. These findings support the use of DCBs as a non-stent-based strategy, particularly valuable in patients with high restenosis risk or anatomical constraints where further stenting is unfavorable.

This is the first large-scale U.S. trial validating the safety and efficacy of coronary DCBs, addressing a long-standing treatment gap for ISR and supporting their regulatory consideration in the United States.

(b) Small Vessel Disease (SVD)

SVD (vessel diameter < 2.75 mm) is associated with higher restenosis and thrombosis risks due to limited luminal area [31].

The BASKET-SMALL 2 trial, a multicenter, open-label, randomized non-inferiority study, evaluated drug-coated balloons (DCB, SeQuent Please) versus second-generation drug-eluting stents (DES, 75% Xience, 25% Taxus Element) in 758 patients with de novo lesions in small coronary arteries (<3 mm diameter) requiring percutaneous coronary intervention [13].

Patients were randomized 1:1 after successful predilatation, ensuring no flow-limiting dissection or residual stenosis > 30%. The primary endpoint was major adverse cardiac events (MACE: cardiac death, non-fatal myocardial infarction, target-vessel revascularization) at 12 months.

The results demonstrated DCB non-inferiority to DES, with MACE rates of 7.5% (DCB) versus 7.3% (DES) (HR 0.97; 95% CI 0.58–1.64; P non-inferiority = 0.037). Individual endpoints showed no significant differences: cardiac death (3.1% vs. 1.3%), myocardial infarction (1.6% vs. 3.5%), and target-vessel revascularization (3.4% vs. 4.5%).

Stent thrombosis (0.8% vs. 1.1%) and major bleeding (1.1% vs. 2.4%) were lower in the DCB group, though not statistically significant.

The tree-year follow up confirmed sustained non-inferiority (MACE: 13% vs. 12%). DCB advantages include shorter dual antiplatelet therapy (DAPT) duration (4 weeks vs. 6–12 months for DES) and no permanent implant, potentially reducing thrombotic risk.

**Figure 5:**
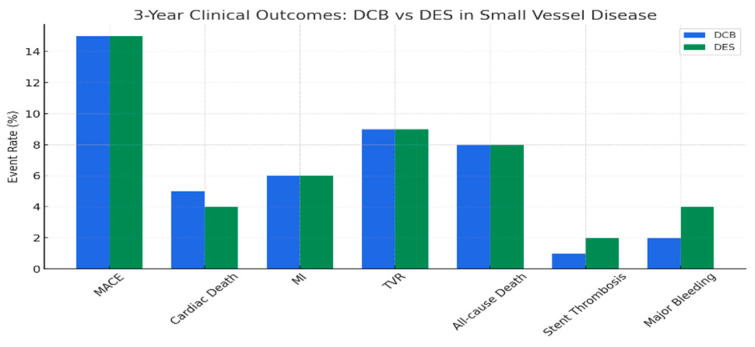
Clinical outcomes at 3 years: DCBs vs. DESs.

The trial suggests DCBs as a viable alternative to DESs in small-vessel disease, particularly for high-bleeding-risk patients, though successful lesion preparation is critical (Figure 5).

In the same spirit, another study, the BIO-RISE CHINA trial, evaluated a novel biolimus-coated balloon (BCB) in patients with small-vessel coronary artery disease [33]. In this randomized study of 212 patients, BCB significantly reduced late lumen loss at 9 months (0.16 mm vs. 0.30 mm, *p* = 0.001) compared to plain balloon angioplasty. Positive vascular remodeling was more frequent with BCB (29.7% vs. 9.8%, *p* = 0.007). At 12 months, target lesion failure and patient-oriented clinical outcomes were numerically lower in the BCB group. This first-in-human study suggests that BCB offers enhanced efficacy and vascular healing in small-vessel PCI.

The RESTORE SVD trial (*n* = 230) corroborated these findings, reporting comparable outcomes in patients treated with DCB vs. DES [34]. There was no significant difference in TLF between the DCB and DES groups (5.2 vs. 3.7%, *p* = 0.75). Imaging studies suggest better endothelial recovery and reduced late inflammation in DCB-treated vessels.

(c) De Novo Lesions

DCBs are increasingly utilized for de novo lesions, particularly in HBR or elderly patients where prolonged DAPT is undesirable [35].

The PICCOLETO II trial (Drug Eluting Balloon Efficacy for Small Coronary Vessel Disease Treatment; NCT03899818) is an international, investigator-driven, multicenter, open-label, randomized controlled trial comparing a paclitaxel-coated drug-coated balloon (DCB, Elutax SV) with an everolimus-eluting stent (EES, Xience) in 232 patients with de novo small-vessel coronary artery disease (SVD, diameter < 3 mm) [14].

Conducted across five European centers from 2015 to 2018, the trial aimed to assess DCB efficacy versus EES in SVD, a challenging subset due to higher restenosis rates. Patients with stable or unstable coronary artery disease scheduled for percutaneous coronary intervention (PCI) were randomized 1:1 (DCB: *n* = 118; EES: *n* = 114) after successful lesion predilatation.

The primary endpoint, in-lesion late lumen loss (LLL) at 6 months, was significantly lower in the DCB group (0.04 mm) compared to the EES group (0.17 mm; P non-inferiority = 0.01, P superiority = 0.03), demonstrating DCB superiority.

At the 3-year follow-up, major adverse cardiac events (MACE: cardiac death, non-fatal myocardial infarction, target lesion revascularization) were less frequent with DCB (10.8%) versus EES (20.8%; *p* = 0.046).

Individual endpoints showed no significant differences, but trends favored DCB: cardiac death (2.5% vs. 3.5%), myocardial infarction (1.7% vs. 4.4%), and target lesion revascularization (6.8% vs. 12.3%).

**Figure 6:**
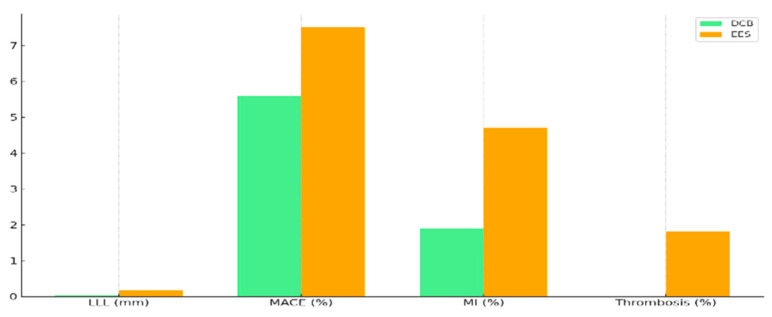
DCB vs. EES outcomes in PICCOLETO II trial.

DCB required shorter dual antiplatelet therapy (DAPT; 30 days for stable disease, 12 months for acute coronary syndrome) compared to EES (6–12 months), potentially reducing bleeding risk (Figure 6).

The trial highlights DCB as a superior alternative to EES in SVD, offering better angiographic outcomes and lower MACE rates, with the advantage of no permanent implant. Further studies powered for clinical endpoints are needed to confirm these findings.

(d) Bifurcation Lesions

Bifurcation lesions pose technical challenges due to complex anatomy and the risk of side branch occlusion. DCBs are effective for side branch treatment after main branch stenting (or in Medina 0-0-1 type bifurcation), preserving vessel anatomy and reducing the need for complex two-stent strategies [36]. Observational data and small RCTs report favorable patency rates (90–95%) and lower MACE with DCB use in side branches [37].

(e) High-Bleeding-Risk (HBR) Patients

HBR patients, including the elderly, those with chronic kidney disease, or recent bleeding, benefit from DCB shorter DAPT requirements (1–3 months vs. 6–12 months for DES) [24]. Also, in the BASKET-SMALL-2 trial, DCBs showed numerically lower rates of major bleeding (2% vs. 4%; HR 0.43; *p* = 0.088) [13].

The ESC 2023 guidelines endorse DCBs for HBR patients, citing reduced bleeding events and comparable efficacy to DESs in ISR and SVD [24].

(f) Potential roles of DCBs

Drug-coated balloons (DCBs) are emerging as a potential therapy for unstable plaques in acute coronary syndromes (ACS) and diffuse coronary artery disease (CAD), offering a stent-free approach to deliver antiproliferative drugs. Their role in these settings is supported by theoretical justifications and limited but growing clinical evidence.

### 4.1. Unstable Plaques (ACS, Ruptured Lesions)

DCBs may stabilize vulnerable plaques by delivering drugs like paclitaxel or sirolimus to inhibit neointimal hyperplasia while avoiding stent-related complications (e.g., thrombosis). The cytostatic mechanism of sirolimus DCBs could reduce inflammation in ACS, theoretically promoting plaque passivation [38].

The PEPCAD NSTEMI trial (2020) evaluated paclitaxel DCBs in non-ST-elevation myocardial infarction (NSTEMI), showing comparable 9-month major adverse cardiac events (MACE) to drug-eluting stents (DESs) (6.7% vs. 7.1%; *p* = 0.85) [39]. Optical coherence tomography (OCT) studies suggest DCBs achieve adequate drug penetration in ruptured plaques, but data remain limited, and lesion preparation is critical to avoid dissections [40].

Emerging evidence supports DCB use in select ACS cases with focal, non-complex lesions post-successful predilatation.

### 4.2. Diffuse Coronary Disease

Diffuse CAD, characterized by long or multifocal lesions, poses challenges for stenting due to increased restenosis and thrombosis risks. DCBs offer a viable alternative, particularly in tandem or hybrid strategies with DES.

The BASKET-SMALL 2 trial (2018) demonstrated DCB non-inferiority in small vessel disease, often diffuse, with MACE rates of 7.5% (DCB) vs. 7.3% (DES) at 12 months [13].

Tandem DCB use (sequential balloon inflations along diffuse segments) or hybrid approaches (DCB for distal segments, DES for proximal) are feasible, especially in high-bleed-risk patients. The HYPER trial (2023) reported a 12-month target lesion revascularization (TLR) rate of 5.2% with hybrid DCB-DES strategies in diffuse CAD [41]. However, rigorous predilatation and imaging (IVUS/OCT) are essential to ensure uniform drug delivery.

DCBs show promise in ACS and diffuse CAD, supported by theoretical anti-inflammatory benefits and early trial data. Tandem or hybrid strategies enhance their applicability, but larger studies are needed to establish efficacy and safety.

Beyond controlled trials, real-world evidence (RWE) from registries provides insights into DCB efficacy and safety, reflecting diverse patient populations and clinical settings.

The PEPPER registry (2021), a multicenter European study, evaluated paclitaxel-coated DCBs in 1024 patients with ISR and de novo lesions. It reported a 12-month major adverse cardiac event (MACE) rate of 8.7%, with target lesion revascularization (TLR) at 5.3%, demonstrating DCB effectiveness in routine practice [42]. For ISR, DCBs showed comparable outcomes to drug-eluting stents (DES), with lower DAPT duration (3 vs. 12 months), benefiting HBR patients.

The EASTBOURNE registry (2023) assessed sirolimus-coated DCBs in 2123 patients across coronary indications, reporting a 9.1% MACE rate and 4.8% TLR at 12 months, supporting their use in SVD and complex lesions [43]. This highlights the growing role of sirolimus DCBs in real-world settings.

In SVD, the BASKET-SMALL 2 registry extension (2020) confirmed DCB non-inferiority to DES, with 3-year MACE rates of 13% (DCB) vs. 12% (DES; *p* = 0.71), highlighting sustained real-world efficacy [44].

For HBR patients, the DEBUT registry (2020) showed that DCBs reduced bleeding events (4.1% vs. 9.2%; *p* = 0.04) compared to bare-metal stents, reinforcing their role in this cohort [19].

Challenges like vessel recoil and geographic mismatch persist, with a 2022 analysis reporting a 6.2% failure rate due to inadequate lesion preparation [45]. Ongoing registries like SIRONA (PAD-focused) continue to explore sirolimus DCBs, suggesting evolving applications [46].

RWE underscores DCBs’ versatility but emphasizes the need for meticulous technique, including predilatation and imaging guidance (IVUS/OCT). These data support DCBs as a viable alternative in real-world PCI, particularly for ISR, SVD, and HBR patients.

## 5. Comparative Efficacy: Paclitaxel vs. Sirolimus

(a) Randomized Trials and Meta-Analyses

Head-to-head comparisons of paclitaxel and sirolimus DCBs are limited but growing. A 2023 meta-analysis (*n* = 3245) found comparable TLF (11.8% vs. 12.1%) and TLR (7.9% vs. 8.2%) rates between paclitaxel and sirolimus DCBs in ISR and SVD [47]. Sirolimus DCBs showed a trend toward lower LLL (0.24 mm vs. 0.31 mm, *p* = 0.06) and improved endothelial healing, as evidenced by optical coherence tomography (OCT) studies [47].

A preclinical study by Aihara et al. (2024) in rabbit iliac arteries demonstrated that paclitaxel-coated DCBs (e.g., AGENT, SeQuent Please NEO) caused greater SMC loss and delayed healing compared to sirolimus-coated DCBs (e.g., MagicTouch), supporting the claim of superior vascular healing for SCBs [38].

The SIRPAC trial (2021) compared sirolimus- and paclitaxel-coated DCBs for coronary in-stent restenosis (ISR), and found no significant difference in major adverse cardiac events (MACE) or target lesion failure (TLF) at 12 months (SCB: 10.2% vs. PCB: 11.5%; *p* = 0.78), suggesting comparable clinical healing [48]. Similarly, the SIBLINT-ISR trial (2025) reported non-inferiority of SCBs for ISR, with no significant difference in late lumen loss (LLL) or clinical outcomes, indicating that while preclinical data favor sirolimus, clinical healing benefits are not consistently superior [47].

The SPACIOUS trial (2025) showed that SCBs were non-inferior to PCBs in coronary bifurcation lesions, with similar diameter stenosis (SCB: 28.1% vs. PCB: 29.3%; *p* = 0.45), further tempering claims of superiority [46].

Sirolimus DCBs are being investigated for both coronary artery disease (CAD) and peripheral artery disease (PAD). The XTOSI trial (2021) evaluated the MagicTouch SCB in PAD (below-the-knee lesions) and reported 100% procedural success and 89.7% freedom from target lesion revascularization (TLR) at 12 months, suggesting potential applicability in complex PAD settings where paclitaxel has concerns (e.g., distal embolization) [49]. In contrast, paclitaxel DCBs have a more established role in PAD, with trials like IN.PACT SFA (2015) demonstrating sustained patency (2-year freedom from TLR: 78.9%) and multiple approved devices (e.g., IN.PACT Admiral) [50]. Sirolimus DCBs require advanced excipient systems due to lower lipophilicity, which may limit their applicability without optimized delivery, as explored in the SELUTION SLR trial (2023) for PAD [51].

A 2024 meta-analysis by Shin et al. found no significant difference in TLF between SCBs and PCBs in coronary PCI (OR 1.01, 95% CI 0.75–1.35), though PCBs showed a slightly larger minimal lumen diameter (MLD) [20].

The ongoing SIRONA trial (protocol 2021, results pending) compares SCBs and PCBs in PAD, but lacks completed data to confirm broader applicability [47]. Safety concerns with paclitaxel in PAD (e.g., FDA’s 2019 mortality signal) have spurred interest in sirolimus, but the SAFE-PAD study (2022) found no mortality difference, reducing the urgency for SCBs [52].

Also, ongoing trials, such as TRANSFORM II, will clarify their role and drug clinical benefits (Paclitaxel vs. Sirolimus) in de novo lesions and complex anatomies [53].

Thus, regarding the differences between sirolimus DCBs and paclitaxel DCBs, things are not fully clarified, but further research should clarify this.

(b) Safety and Healing

Paclitaxel DCBs’ association with increased morbidity in peripheral artery disease raised concerns, but coronary DCB studies have not substantiated these risks [54]. Sirolimus DCBs demonstrate superior endothelialization and reduced inflammatory infiltrates in preclinical models and OCT studies, potentially lowering long-term complications. The PEPPER registry (*n* = 816) reported low MACE rates (6.5%) with sirolimus DCBs in ISR, supporting their safety [55].

## 6. Technical Considerations

Optimal DCB outcomes require meticulous lesion preparation and procedural adherence.

Effective lesion preparation is critical for successful drug-coated balloon (DCB) use in percutaneous coronary intervention (PCI), ensuring optimal drug delivery and minimizing complications. Inadequate preparation is a primary cause of DCB failure, leading to suboptimal outcomes such as residual stenosis or dissection [56].

Key components include predilatation, use of semi-compliant or scoring balloons, and imaging guidance with intravascular ultrasound (IVUS) or optical coherence tomography (OCT).

### 6.1. Predilatation

Predilatation with a semi-compliant balloon is essential to achieve a residual stenosis < 30% and no flow-limiting dissection before DCB deployment. The BASKET-SMALL 2 trial (2018) emphasized that successful predilatation in small vessel disease (SVD) was a prerequisite for DCB non-inferiority to drug-eluting stents (DESs), with MACE rates of 7.5% (DCB) versus 7.3% (DES) at 12 months [13].

Inadequate predilatation increases the risk of uneven drug transfer and recoil, compromising efficacy [56].

It is very important that significant dissections do not occur, because flow-limiting dissections (type C or greater) increase failure risk [54]. Also, great care must be taken to achieve TIMI 3 flow and avoid a geographic mismatch [57].

### 6.2. Semi-Compliant and Scoring Balloons

Semi-compliant balloons allow for controlled vessel expansion, minimizing trauma. Scoring balloons, with microblades or wires, are particularly effective for calcified or fibrotic lesions, enhancing plaque modification.

The ISAR-DESIRE 3 trial (2015) showed that scoring balloons improved DCB outcomes in in-stent restenosis (ISR) by ensuring uniform vessel preparation, reducing late lumen loss (LLL) to 0.05 mm versus 0.21 mm with standard balloons [10].

### 6.3. Imaging Guidance (IVUS/OCT)

IVUS and OCT provide detailed lesion characterization, guiding optimal balloon sizing and detecting dissections or malapposition. OCT, with higher resolution, is particularly valuable in ISR and SVD, identifying plaque morphology and ensuring adequate expansion.

The RESTORE trial (2018) reported that OCT-guided DCB procedures reduced LLL (0.03 mm) compared to angiography alone [34]. Imaging ensures precise preparation, critical for drug penetration and vessel healing [42].

Inadequate lesion preparation, such as insufficient dilatation or untreated dissections, is a major cause of DCB failure, leading to restenosis or thrombosis. Rigorous preparation protocols enhance DCB efficacy, particularly in ISR, SVD, and high-bleed-risk patients.

### 6.4. Role of Coronary Physiology in DCB Angioplasty

Coronary physiology, assessed by fractional flow reserve (FFR) or instantaneous wave-free ratio (iFR), plays a pivotal role in guiding drug-coated balloon (DCB) angioplasty, optimizing patient selection and post-percutaneous coronary intervention (PCI) outcomes [2,58,59].

DCBs deliver antiproliferative drugs without a permanent stent, requiring precise lesion assessment to ensure functional improvement and prevent restenosis. FFR and iFR provide objective measures of lesion significance and post-PCI vessel patency, enhancing DCB efficacy in settings like in-stent restenosis (ISR) and small vessel disease (SVD).

### 6.5. FFR in DCB Angioplasty

FFR, measuring the pressure gradient across a stenosis, guides DCB use by confirming hemodynamic significance (FFR ≤ 0.80) before PCI. Post-PCI, FFR verifies functional success, with values > 0.85 indicating optimal drug delivery and minimal residual ischemia.

The FFR-SEARCH registry (2019) showed that post-PCI FFR > 0.85 in DCB-treated ISR lesions correlated with lower target lesion revascularization (TLR) rates (4.2% vs. 9.8%; *p* = 0.03) at 12 months [60]. FFR-guided DCB use ensures targeted therapy, avoiding unnecessary interventions in non-ischemic lesions [13].

### 6.6. iFR in DCB Angioplasty

iFR, a non-hyperemic index, simplifies assessment by avoiding adenosine, making it practical for post-PCI evaluation.

The DEFINE-PCI trial (2020) demonstrated that iFR > 0.89 post-DCB PCI predicted low MACE rates (5.1% vs. 12.3% for iFR ≤ 0.89; *p* = 0.02) in SVD [59]. iFR’s ease of use enhances its applicability in complex lesions, such as bifurcations, where DCBs are increasingly used [46].

### 6.7. Post-PCI Role

Post-PCI physiology assessment ensures adequate lesion preparation and drug penetration, critical for DCB success. Inadequate preparation or residual ischemia (low FFR/iFR) is a major cause of DCB failure.

OCT-guided FFR/iFR integration further refines outcomes by identifying dissections or malapposition [40].

Physiology-guided DCB therapy optimizes functional results, particularly in ISR and SVD, reducing adverse events.

The successful use of drug-coated balloons (DCBs) in percutaneous coronary intervention (PCI) hinges on operator technique, experience, and adjunctive tools like intravascular imaging.

The learning curve for DCB angioplasty is steep due to the need for meticulous lesion preparation, precise balloon sizing, and optimal drug delivery, differing from drug-eluting stent (DES) procedures. Inadequate technique increases risks of vessel recoil, geographic mismatch, and suboptimal outcomes, particularly in in-stent restenosis (ISR) and small vessel disease (SVD) [56].

Operators must master balloon sizing (1:1 vessel ratio) and inflation timing (30–60 s) to optimize drug transfer, particularly for paclitaxel or sirolimus DCBs [38].

LLL, a measure of neointimal hyperplasia post-PCI, is a critical metric for DCB efficacy. The RESTORE trial (2018) reported an LLL of 0.03 mm in DCB-treated ISR, comparable to DESs, but inexperienced operators saw a higher LLL (0.15 mm) due to poor preparation [34].

The catch-up phenomenon, where initial lumen gains diminish over time, is less pronounced with DCBs than early-generation DESs, but can occur if drug delivery is uneven. A 2020 meta-analysis noted a 5.8% catch-up rate in DCB-treated lesions, linked to inadequate predilatation or a geographic mismatch [57].

Late lumen enlargement, observed in some DCB cases, reflects positive vessel remodeling due to reduced inflammation. However, potential late failures (e.g., restenosis) necessitate follow up with angiography or physiology (FFR/iFR) at 6–12 months. OCT detects neointimal changes, aiding in identifying late failures [3]. Concerns about sirolimus DCBs include lower tissue retention, potentially increasing late restenosis, requiring further study [38].

Thus, the DCB learning curve demands technical proficiency and imaging guidance to minimize LLL and catch-up. Regular follow up with imaging and physiology ensures the early detection of late failures, optimizing long-term outcomes.

## 7. Discussion

DCBs represent a paradigm shift in PCI, offering a stentless alternative that addresses the limitations of DESs while maintaining comparable efficacy. Their ability to deliver antiproliferative drugs without permanent implants reduces risks of stent thrombosis, neoatherosclerosis, and chronic inflammation, which are particularly relevant in complex lesions and HBR patients [9,24]. The shorter DAPT requirement is a critical advantage, as prolonged DAPT increases bleeding risks, particularly in elderly or comorbid patients [10]. Trials like AGENT IDE and BASKET-SMALL 2 underscore DCBs efficacy in ISR and SVD, with outcomes rivaling or surpassing DES [13,32].

The transition from paclitaxel to sirolimus DCBs marks a significant advancement. The sirolimus cytostatic mechanism and improved healing profile address concerns about paclitaxel’s cytotoxicity, potentially broadening DCB indications [11]. However, sirolimus DCBs require sophisticated excipient systems, which increase production complexity and cost [27].

Sirolimus-coated balloons (SCBs) may offer advantages in vascular healing due to their cytostatic mechanism, which is less cytotoxic than paclitaxel, potentially reducing vascular toxicity.

Sirolimus inhibits the mTOR pathway, arresting the cell cycle in the G1 phase, which is cytostatic and promotes anti-inflammatory effects, potentially leading to better endothelialization and less medial smooth muscle cell (SMC) loss compared to paclitaxel, which stabilizes microtubules, causing G2/M phase arrest and cytotoxicity.

While preclinical data (e.g., Aihara et al.) suggest better histological healing with SCBs, clinical trials (SIRPAC, SIBLINT-ISR, SPACIOUS) show non-inferiority rather than superiority in clinical endpoints, limiting the strength of the “superior healing” claim.

Sirolimus DCBs show promise in niche applications (e.g., BTK PAD, ISR), but paclitaxel DCBs have a more established evidence base and broader regulatory approval, particularly in PAD. Sirolimus’ applicability is constrained by delivery challenges, requiring further optimization.

Large-scale, head-to-head randomized controlled trials (RCTs) comparing SCBs and PCBs across diverse indications are limited, and ongoing trials like SIRONA, TRANSFORM II and others, are yet to provide definitive data.

On the other hand, DCBs have some limitations such as vessel recoil, geographic mismatch, and lack of mechanical scaffolding in unstable or calcified lesions, which restrict the efficacy and applicability of DCBs.

DCBs rely on vessel wall drug delivery without a permanent scaffold, making them susceptible to elastic recoil, particularly in elastic or fibrotic vessels. The RESTORE trial (2018) reported that inadequate predilatation led to recoil in 8.3% of DCB-treated ISR cases, increasing late lumen loss (LLL) to 0.12 mm compared to 0.03 mm with optimal preparation [34].

A geographic mismatch, where the DCB-treated segment does not fully cover the lesion, can result in untreated areas, leading to restenosis. A 2020 meta-analysis found that geographic mismatch contributed to a 6.5% target lesion revascularization (TLR) rate in DCB procedures, especially in complex lesions [57]. Intravascular ultrasound (IVUS) or optical coherence tomography (OCT) can mitigate this by ensuring precise lesion coverage [40].

Unlike drug-eluting stents (DESs), DCBs provide no structural support, posing challenges in unstable or calcified lesions. These lesions, common in elderly patients, resist adequate dilatation, reducing drug penetration. The ISAR-DESIRE 3 trial (2015) showed higher TLR (9.2%) in calcified ISR treated with DCBs versus DES (4.5%; *p* = 0.04) [10]. Scoring balloons or an atherectomy may improve outcomes but add procedural complexity.

Vessel recoil, geographic mismatch, and lack of scaffolding limit DCB use in complex, unstable, or calcified lesions. Imaging-guided preparation and hybrid strategies may address these challenges, but DESs remain superior in such cases.

Challenges remain, including optimizing drug formulations for calcified lesions and ensuring uniform drug delivery in tortuous vessels [44].

The role of AI in lesion characterization and procedural planning is an exciting frontier, with potential to enhance precision and outcomes [61].

Additionally, the economic impact of DCBs, particularly in resource-limited settings, warrants further study, as their upfront costs may be offset by reduced long-term complications and DAPT duration [55].

## 8. Future Perspectives

The future of DCBs is promising, with several avenues for innovation:

- Dual-Drug DCBs: Combining paclitaxel and sirolimus could leverage synergistic effects, potentially reducing restenosis rates further [12].
- Ultra-Thin Balloons: These enhance deliverability in complex anatomies, minimizing vessel trauma [27].
- Novel Coatings: Biodegradable matrices and nanoparticle-based systems may improve drug elution and reduce inflammation [12].
- AI Integration: AI-driven algorithms for lesion characterization, balloon sizing, and patient selection could optimize outcomes [61].
- Expanded Indications: Trials like TRANSFORM II and SELUTION ISR are exploring DCBs in acute coronary syndromes and chronic total occlusions [51,53].

Advancements in imaging, such as high-resolution OCT and AI-enhanced IVUS, will further refine procedural precision, ensuring optimal lesion preparation and DCB deployment [62]. As evidence accumulates, DCBs may become the preferred strategy for a broader range of coronary lesions, particularly in HBR and complex anatomy patients.

## 9. Conclusions

Drug-coated balloons (DCBs) represent a major innovation in percutaneous coronary intervention (PCI), providing an effective, stent-free approach to deliver antiproliferative drugs, reducing neointimal hyperplasia while minimizing complications like stent thrombosis.

Because of their capacity to deliver antiproliferative agents locally, without the long-term presence of a metallic scaffold, has redefined treatment strategies for in-stent restenosis (ISR), small vessel disease (SVD), de novo lesions, and bifurcation segments, reducing the incidence of late and very late stent-related complications, such as thrombosis and neoatherosclerosis [24]. By eliminating permanent implants, DCBs allow for shorter durations of dual antiplatelet therapy (DAPT), a critical advantage in high-bleeding-risk (HBR) patients.

Recent evidence suggests that sirolimus-based DCBs may provide superior vascular healing and broader applicability compared to paclitaxel-based DCBs, but further research must clarify this.

However, optimal results with DCBs are highly dependent on meticulous lesion preparation, appropriate vessel sizing, and the use of intracoronary imaging modalities such as IVUS or OCT to guide therapy [13].

As the field of interventional cardiology continues to evolve, the role of DCBs is likely to expand. Advances in drug release technologies, balloon material engineering, and integration of artificial intelligence for real-time decision-making are expected to further enhance the safety, efficacy, and precision of DCB-based PCI.

The next steps include larger randomized trials comparing sirolimus vs. paclitaxel DCBs, such as the ongoing SIRONA trial, to establish superiority in coronary and peripheral applications [21].

Standardizing lesion preparation protocols and integrating physiology (FFR/iFR) post-PCI will optimize the outcomes.

Research should focus on DCBs in acute coronary syndromes (ACSs) and diffuse CAD, exploring tandem or hybrid strategies. Clinically, operators need training to master the DCB learning curve, emphasizing imaging-guided techniques to reduce recoil and geographic mismatch.

In this context, DCBs are not merely an alternative to drug-eluting stents; they are emerging as a foundational strategy in the management of coronary artery disease, especially in anatomically or clinically challenging scenarios.

Ultimately, the continued adoption of DCBs will rely on robust clinical evidence, practitioner familiarity with technique-specific protocols, and thoughtful patient selection. When applied appropriately, DCBs offer a tailored, minimally invasive, and durable solution that aligns with the evolving goals of modern coronary revascularization.

## Figures and Tables

**Table 1:** Comparison of paclitaxel and sirolimus DCB properties.

Property	Paclitaxel	Sirolimus
Lipophilicity	High (logP 3.96)	Moderate (logP 2.5)
Mechanism	Microtubule stabilization	mTOR inhibition
Tissue Retention	Long (weeks)	Shorter (requires sustained delivery)
Excipient Examples	Shellac, urea, iopromide	BTHC, PLGA, phospholipids
Healing Profile	Delayed endothelialization	Faster endothelialization
Inflammatory Response	Moderate	Low
